# Carcinome rénal à cellules chromophobes: à propos de quatre cas et revue de la literature

**DOI:** 10.11604/pamj.2015.22.123.6741

**Published:** 2015-10-12

**Authors:** Yddoussalah Othmane, Lahyani Mounir, Karmouni Tarik, Elkhader Khalid, Koutani Abdellatif, Ibn Attya Andaloussi Ahmed

**Affiliations:** 1 Centre Hospitalier et Universitaire Ibn Sina, Hôpital Ibn Sina, Service d'Urologie B, Rabat, Maroc

**Keywords:** Carcinome à cellules chromophobes, néphrectomie, pronostic, Chromophobe renal cell carcinoma:, nephrectomy, prognosis

## Abstract

Nous rapportons dans cet article trois cas de carcinome rénal à cellules chromophobes. Le carcinome chromophobe se voit essentiellement à la cinquième décennie et représente 5% des tumeurs rénales. Il existe deux sous types de cancer à cellules chromophobes: le type clair (70%) et le type éosinophile. La variante à cellules éosinophiles doit être distinguée de l'oncocytome. Ce dernier se caractérise par une cicatrice fibreuse centrale d'aspect stellaire. Le carcinome chromophobe et l'oncocytome peuvent même coexister dans le cadre du syndrome de Birt-Hogg-Dubé. Certaines tumeurs appelées hybrides partagent des caractéristiques architecturales et cytologiques de ces deux tumeurs. Le pronostic du carcinome chromophobe est favorable. Il est le plus souvent limité au rein et de bas grade nucléaire. Il semblerait donc licite dans les cas ou un examen extemporané mettrait en évidence une tumeur chromophobe, de limiter l'intervention à une néphrectomie partielle si elle est techniquement réalisable.

## Introduction

En France en 2011, l'Institut National du Cancer rapporte 1000 nouveaux cas de cancer du rein, soit près de 3% de l'ensemble des cancers, ceci correspondant au 6^éme^ rang par incidence et au 7^éme^ rang de mortalité (2,6% des décès par cancer). Les carcinomes à cellules chromophobes représentent 3,6 à 10,4% des pièces de néphrectomie pour cancer [1] et correspondent au troisième sous-type histologique en fréquence, après les carcinomes à cellules claires et les carcinomes papillaires. C'est Thoenes et al qui a décrit pour la première fois les carcinomes à cellules chromophobes, en 1985 [2], ils sont généralement diagnostiqués durant la cinquième décade. Contrairement au carcinome à cellules claires, les carcinomes à cellules chromophobes sont d'excellent pronostic. Nous avons revu quatre cas de cancer chromophobe du rein dans le but d’étudier ses caractéristiques cliniques, radiologiques, anatomo-pathologiques et évolutives.

## Patient et observation

### Cas N° 1

**Figure 1:**
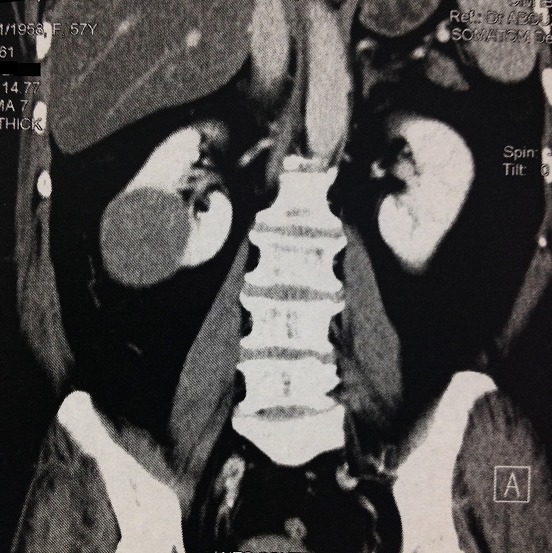
Coupe tomodensitométrique frontale mettant en évidence la masse polaire inferieur du rein droit mesurant 60x50 mm de taille

Mme Z.D, âgée de 58 ans, ayant comme antécédent pathologique une fistule anale opérée. Elle se plaignait depuis six mois de lombalgies droites irradiant vers la cuisse gauche dans un contexte d'anorexie et amaigrissement, sans trouble urinaire, digestif associé. A l'examen clinique, la patiente a été apyrétique. Ses conjonctives ont été normalement colorées, et son abdomen souple. Les aires ganglionnaires ont été libres. La tomodensitométrie a objectivé un processus lésionnel polaire inferieur du rein droit, de 7 cm de grand axe, dont la densité a été tissulaire, se rehaussant après injection du produit de contraste de façon hétérogène. Il n'y a pas eu d'envahissement de la veine rénale ni de la veine cave inferieur (Figure 1). Deux adénopathie latero aortiques gauches de 6 mm étaient visible. Sur le plan biologique, la patiente a eu un taux d'hémoglobine à 13 g/dl, des leucocytes à 5400/mm3, une fonction rénale normale avec une créatininémie à 9,32 mg/L. La patiente a été opérée par voie sous-costale droite. Vu la taille de la Tumeur et ses rapports avec le pédicule rénal, nous avons réalisé une néphrectomie totale droite. Les suites opératoires ont été simples. À l'examen macroscopique à la coupe, la masse a été molle, d'aspect encapsulée et de couleur beige. L'examen microscopique a mis en évidence une prolifération tumorale faite de nappes, de travées et de lobules de cellules tumorales. Les cellules tumorale sont de grand tailles et d aspect végétal a cytoplasme éosinophile et aux noyaux chiffonnés entoures d un halo clair. Les noyaux sont ronds monomorphes et atypiques. La capsule, les vaisseaux du hile et la recoupe urétérale sont sains. Cette analyse histologique a conclu à un carcinome chromophobe du rein grade IV de Furhman.

### Cas N° 2

**Figure 2:**
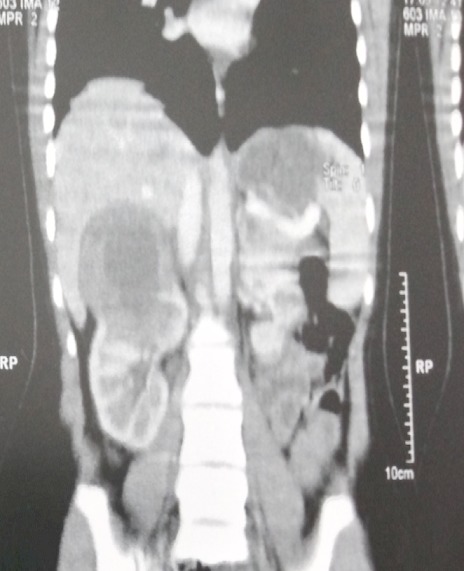
TDM abdominale montrant processus tissulaire de 7 cm de grand axe polaire supérieure hypodense. Cette lésion est faiblement rehaussée après injection de produit de contraste

**Figure 3:**
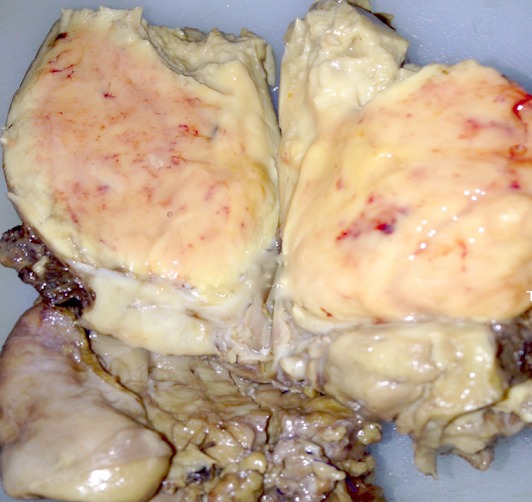
Volumineuse tumeur dont la tranche de section est de couleur jaune rose, avec quelques foyers hémorragiques (aspect macroscopique)

**Figure 4:**
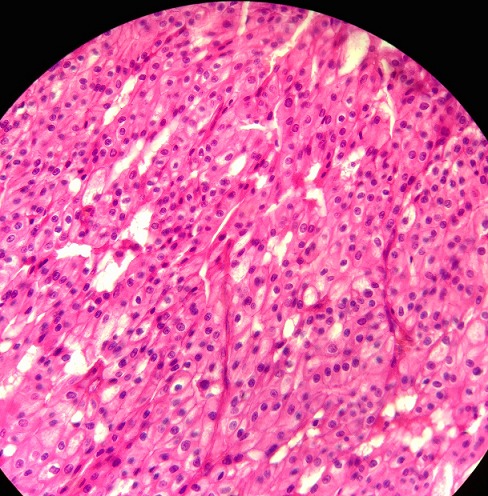
Cancer chromophobe du rein du rein (400x, H&E)

Mme R.K âgée de 25 ans, sans antécédents particuliers, a présenté des douleurs de l'hypochondre droit. L’échographie abdominale a montré La présence d'une masse de 7,5 cm de diamètre d'allure tissulaire aux dépens du rein droit. La fonction rénale était normale. Le scanner abdominal a montré un processus tissulaire de 7 cm de grand axe polaire supérieur hypodense. Cette lésion est faiblement rehaussée après injection de produit de contraste (Figure 2). Le rein controlatéral était normal. Nous avons réalisé une néphrectomie totale élargie droite. L'examen anatomopathologique de la pièce opératoire a montré la présence d'une Volumineuse tumeur dont la tranche de section est de couleur jaune rose, avec quelques foyers hémorragiques (Figure 3). A L'examen histologique, les coupes analysées montrent une large prolifération carcinomateuse au sein du parenchyme rénal, fait de nappes de travées et de lobules de cellule tumoral de grand taille d'aspect végétal a cytoplasme éosinophile et aux noyaux entourés d'un halo clair. Les noyaux sont ronds monomorphes discrètement atypiques. On n'a pas vu de composante sarcomatoide. Le tout est en faveur d'un carcinome à cellules chromophobes rénales (Figure 4).

### Cas N° 3

**Figure 5:**
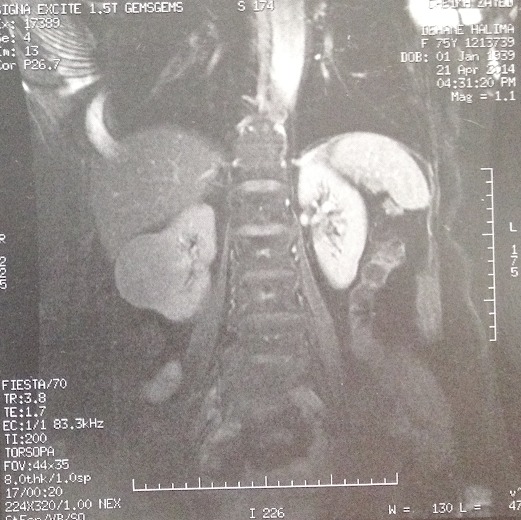
Aspect IRM montrant un processus tissulaire bien limité, mesurant 7 cm de grand axe. Ce processus est peu rehaussé par le gadolinium

Mme F.B 72 ans, obèse, traitée pour kyste hydatique pulmonaire il y a 10 ans et ayant consulté pour des lombalgies droites évoluant depuis un an. L'examen clinique était normal. L’échographie rénale a mis en évidence une masse tissulaire rénale droite. L'uro-scanner a objectivé une masse médio-rénal droite de 6 cm de grand axe, hypodense, se rehaussant faiblement après injection du produit de contraste, avec suspicion de thrombus tumoral de la veine cave inferieur. Pour mieux évaluer le thrombus veineux, nous avons réalisé une IRM abdominale qui a montré un processus tissulaire bien limité en hypo signal T1, mesurant 7 cm de grand axe. Ce processus est peu rehaussé par le gadolinium. Le carrefour réno-cave est perméable (Figure 5). Une néphrectomie droite par voie antérieure sous-costale a également été réalisée. L'histologie de la pièce a montre une masse de consistance molle et de couleur beige arrivant au contact de la capsule rénale.

### Cas N° 4

**Figure 6:**
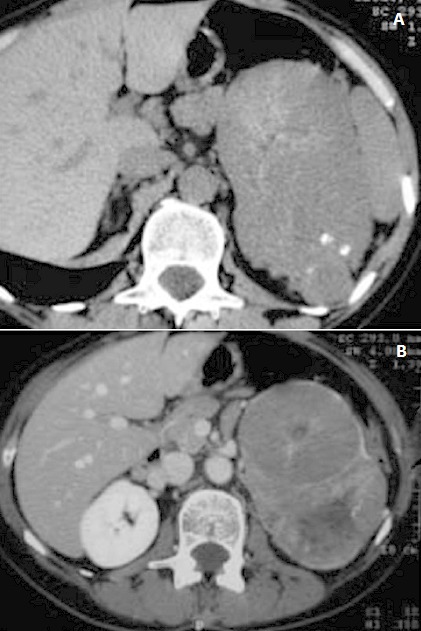
TDM sans puis après injection de contraste: masse de densité tissulaire, polylobée, relativement homogène sans injection, contenant des calcifications, dont le rehaussement fait apparaître des zones nodulaires dont certaines ont un caractère stellaire

Mme S.S, 51 ans, sans antécédents notables, accuse des lombalgies gauches depuis 6mois. La fonction rénale est normale. L’échographie retrouve une masse tissulaire hypoéchogène hétérogène occupant la moitié supérieure du rein gauche. L'examen TDM, montre un volumineux processus tissulaire polylobé, bien limité, avec quelques calcifications, de 8cm de grand axe, et dont le prise de contraste après injection fait apparaître sur certaines portions un caractère stellaire (Figure 6). Une néphrectomie gauche élargie a été réalisée. La pièce pesait 400 g et mesurait 15×8×5 cm. Il existait a la coupe une volumineuse masse tissulaire polaire, de consistance moelle, de couleur beige avec des remaniements nécrotico-hémorragiques. Microscopiquement, la tumeur était compacte organisée en massifs faits de cellules de taille moyenne à grande circonscrites par une membrane cytoplasmique bien visible. Les noyaux étaient tantôt arrondis, tantôt fripés et pourvus d'un nucléole visible au fort grandissement. Le tout est en faveur d'un carcinome à cellules chromophobes rénal grade II de furhman. Toutes les patientes ont été revues en consultation à un mois, six mois et un an; leur examen clinique a été normal et la tomodensitométrie thoraco-abdominal réalisée après un an n'a pas mis en évidence de récidive locale ni de métastases à distance.

## Discussion

En 1985, Thoenes et coll. de Mayence ont décrit une nouvelle entité appelée carcinome à cellules chromophobes. Elle représente entre 3.6% a 10.4% de tous les cancers du rein [3]. Le terme chromophobe a été utilisé par opposition au cancer classique anciennement dit chromophile. C'est une tumeur qui atteint préférentiellement le sexe féminin à un âge plus jeune que les autres types de carcinome [4]. Une récente étude a comparé les facteurs de risque au sous-type histologique des carcinomes rénaux, et confirme que la consommation tabagique et l'obésité sont des facteurs de risques de cancer chromophobe du rein [5]. Les carcinomes rénaux à cellules chromophobes sont souvent cliniquement asymptomatiques et de découverte fortuite au décours d'une imagerie abdominale pour diverses indications. Les signes d'appel cliniques sont l'hématurie macroscopique, la douleur lombaire et le syndrome de masse dans la fosse lombaire. La présence de signes cliniques est souvent synonyme de tumeur plus volumineuse et évoluée. En tomodensitométrie, les carcinomes rénaux à cellules chromophobes prennent le contraste de façon homogène alors que dans les carcinomes à cellules claires, les carcinomes papillaires et les carcinomes des tubes collecteurs la prise de contraste est périphérique et hétérogène [6]. L'imagerie par résonnance magnétique retrouve globalement les mêmes caractéristiques que les carcinomes à cellules claires, à savoir une lésion hyposignal en T1. Pour ce qui est des critères anatomopathologiques, ils se sont précisés depuis la première observation de Thoenes et al. Sur le plan macroscopique, il s'agit de tumeur unifocale, bien limitée mais non encapsulée, parfois lobulée, de couleur grise, beige à brune ou jaune. Les remaniements hémorragiques ou nécrotiques sont observés dans environ 15% des cas [3, 7]. Sur le plan microscopique, l'architecture tumorale est le plus souvent faite de larges alvéoles de cellules séparées par de fines cloisons fibrovasculaires, mais aussi de nids de cellules, de travées, de nappes solides, de tubules ou de papilles. Dans les carcinomes à cellules claires, 2 types de cellules tumorales sont décrites: les cellules d'aspect « classique » et les cellules d'aspect « éosinophile». Ainsi trois variantes de carcinome chromophobe sont décrites: la variante classique, possédant plus de 80% de cellules classiques; la variante éosinophile, possédant plus de 80% de cellules éosinophiles; la variante mixte, possédant plus de 20% des deux types de cellules. Plus récemment, une équipe japonaise a proposé une nouvelle variante de carcinome chromophobe appelé variante « oncocytique » qui présente une cytologie d'oncocytome, avec de grandes cellules éosinophiles et des noyaux de petite taille, arrondis, mais des caractéristiques immunohistochimiques et cytogénétiques de carcinome chromophobe [8]. Cette variante n'est cependant pas en encore reconnue par les sociétés internationales d'uropathologie. En cas de doute, la coloration de Hale permet d'affirmer le diagnostic de carcinome chromophobe puisqu'elle marque de manière caractéristique le cytoplasme des cellules, qu'elles soient claires ou éosinophiles [9]. Elle est positive dans 10% des cas dans toutes les séries étudiées. Jusqu’à la fin de l'année 2013, le grade nucléaire de Führman était appliqué aux carcinomes rénaux à cellules chromophobes, mais la conférence de consensus de l'International Society of Urological Pathology (ISUP) de Vancouver de 2013 a recommandé de ne plus l'utiliser pour ce type de carcinome [10]. En microscopie électronique, les cellules possèdent de nombreuses microvésicules intra cytoplasmiques spécifiques dont l'origine, discutée, est probablement mitochondriale [2]. Le principal diagnostic différentiel du carcinome chromophobe surtout à cellules éosinophiles est l'oncocytome. Les deux types peuvent coexister dans le cadre du syndrome de Birt-Hogg-Dubé une génodermatose autosomique dominante; cliniquement, ce syndrome se manifeste par des tumeurs cutanées bénignes, des kystes pulmonaires associés à des pneumothorax récidivants et des tumeurs rénales, souvent bilatérales et multiples [11]. 50% de ces tumeurs sont représentées par des tumeurs hybrides, tumeur associant un contingent de cellules chromophobes et un second contingent de cellules typiques d'oncocytome. La particularité majeure du cancer chromophobe est la rareté des formes métastatiques. Seul deux cas d'atteinte ganglionnaire synchrone ont été rapportés dans la littérature. De même, un seul cas de métastase synchrone a été décrit: il s'agissait d'un volumineux cancer chromophobe à contingent sarcomateux associé à une localisation pulmonaire. Il semble donc que le cancer chromophobe soit moins souvent diagnostiqué à un stade métastatique que les autres types de cancer du rein, qui s'accompagnent, tous types confondus, de métastases synchrones dans 11 à 28% des cas [12]. Le pronostic du carcinome chromophobe est favorable. Il est le plus souvent limité au rein (stades pT1 et pT2) et de bas grade nucléaire. Différents facteurs pronostiques indépendants liés à la survie sans progression ou lié à la survie spécifique de la maladie ont été rapportés par de nombreux auteurs. Il s'agit du stade tumoral, d'une taille tumorale supérieure à 7cm, de la présence de nécrose tumorale et la présence d'un contingent sarcomatoide [3, 7]. Pour Przybycin et *al*, les emboles tumoraux seraient un facteur de moins bon pronostic [12]. Dans la grande majorité des cas, les carcinomes rénaux à cellules chromophobes sont découverts à un stade précoce et localisé de la maladie (pT1 ou pT2). Il semblerait donc licite, dans les cas où un examen extemporané mettrait en évidence une tumeur chromophobe, et en l'absence d'impossibilité technique, de limiter l'intervention à une néphrectomie partielle. Cependant, l'existence de formes tumorales mixtes associant cancer chromophobe et carcinome à cellules claires [1] doit inciter à la prudence. En effet, la méconnaissance d'un contingent de cancer à cellules conventionnelles lors de l'examen extemporané exposerait au risque de récidive locale ou de métastases. Malgré les avancées thérapeutiques, il n'existe aujourd'hui toujours pas de consensus quant à la prise en charge thérapeutique des formes métastatiques du carcinome chromophobe. Une récente étude de cas a rapporté une survie sans progression de 20 mois sous temsirolimus chez une patiente de 36 ans avec un carcinome chromophobe métastatique, après échec du sunitinib [13].

## Conclusion

Le carcinome chromophobe est une variante relativement rare du carcinome rénal. Il s'agit en général de tumeurs peu agressives, de stade limité et de bas grade. Les formes métastatiques ou récidivantes sont exceptionnelles. De plus grandes études sont nécessaires pour améliorer les outils diagnostiques et pronostiques du carcinome rénale cellule à chromophobe. La néphrectomie total reste le traitement standard de référence, mais le bon pronostic du cancer à cellules chromophobes pourrait justifier une chirurgie rénale conservatrice.
